# Horizon scanning for invasive alien species with the potential to threaten biodiversity in Great Britain

**DOI:** 10.1111/gcb.12603

**Published:** 2014-05-19

**Authors:** Helen E Roy, Jodey Peyton, David C Aldridge, Tristan Bantock, Tim M Blackburn, Robert Britton, Paul Clark, Elizabeth Cook, Katharina Dehnen-Schmutz, Trevor Dines, Michael Dobson, François Edwards, Colin Harrower, Martin C Harvey, Dan Minchin, David G Noble, Dave Parrott, Michael J O Pocock, Chris D Preston, Sugoto Roy, Andrew Salisbury, Karsten Schönrogge, Jack Sewell, Richard H Shaw, Paul Stebbing, Alan J A Stewart, Kevin J Walker

**Affiliations:** 1 Centre for Ecology & HydrologyWallingford, OX10 8BB, UK; 2 Aquatic Ecology Group, Department of Zoology, University of CambridgeCambridge, CB2 3EJ, UK; 3 British Bugs101 Crouch Hill, London, N8 9RD, UK; 4 Institute of Zoology, Zoological Society of LondonRegent's Park, London, NW1 4RY, UK; 5 Centre for Invasion Biology, Department of Botany and Zoology, Stellenbosch UniversityStellenbosch, South Africa; 6 University of BournemouthPoole, BH12 5BB, UK; 7 Aquatic Invertebrates Division, Department of Life Sciences, The Natural History MuseumCromwell Road, London, SW7 5BD, UK; 8 Scottish Marine InstituteOban, Argyll, PA37 1QA, UK; 9 Centre for Agroecology and Food Security, Coventry UniversityPriory St, Coventry, CV1 5FB, UK; 10 PlantLife, Uned 14Llys Castan, Parc Menai, Bangor, LL57 4FD, UK; 11 APEM Ltd., The Technopole CentreMidlothian, EH26 0PJ, UK; 12 Department of Environment, Earth and Ecosystems, The Open UniversityWalton Hall, Milton Keynes, MK7 6AA, UK; 13 Marine Organism Investigations KillaloeCo Clare, Ireland; 14 British Trust for OrnithologyThetford, IP24 2PU, UK; 15 Animal Health and Veterinary Laboratories AgencySand Hutton, York, YO41 1LZ, UK; 16 RHS Garden WisleyNr Woking, Surrey, GU23 6QB, UK; 17 The Marine Biological Association of the United Kingdom, The LaboratoryCitadel Hill, Plymouth, Devon, PL1 2PB, UK; 18 CABI E-UK Bakeham LaneEgham, Surrey, TW20 9TY, UK; 19 Centre for Environment, Fisheries and Aquaculture ScienceBarrack Road, The Nothe, Weymouth, Dorset, DT4 8UB, UK; 20 School of Life Sciences, University of SussexFalmer, Brighton, BN1 9QG, UK; 21 Botanical Society of Britain and Ireland, Natural History MuseumCromwell Road, London, SW7 5BD, UK

**Keywords:** biodiversity impacts, consensus approach, freshwater, horizon scanning, invasive alien species, marine, terrestrial

## Abstract

Invasive alien species (IAS) are considered one of the greatest threats to biodiversity, particularly through their interactions with other drivers of change. Horizon scanning, the systematic examination of future potential threats and opportunities, leading to prioritization of IAS threats is seen as an essential component of IAS management. Our aim was to consider IAS that were likely to impact on native biodiversity but were not yet established in the wild in Great Britain. To achieve this, we developed an approach which coupled consensus methods (which have previously been used for collaboratively identifying priorities in other contexts) with rapid risk assessment. The process involved two distinct phases:

1. Preliminary consultation with experts within five groups (plants, terrestrial invertebrates, freshwater invertebrates, vertebrates and marine species) to derive ranked lists of potential IAS.
2. Consensus-building across expert groups to compile and rank the entire list of potential IAS.

Five hundred and ninety-one species not native to Great Britain were considered. Ninety-three of these species were agreed to constitute at least a medium risk (based on score and consensus) with respect to them arriving, establishing and posing a threat to native biodiversity. The quagga mussel, *Dreissena rostriformis bugensis*, received maximum scores for risk of arrival, establishment and impact; following discussions the unanimous consensus was to rank it in the top position. A further 29 species were considered to constitute a high risk and were grouped according to their ranked risk. The remaining 63 species were considered as medium risk, and included in an unranked long list. The information collated through this novel extension of the consensus method for horizon scanning provides evidence for underpinning and prioritizing management both for the species and, perhaps more importantly, their pathways of arrival. Although our study focused on Great Britain, we suggest that the methods adopted are applicable globally.

## Introduction

Invasive alien species (IAS; synonyms include nonindigenous, nonnative and exotic) are considered to be one of the greatest threats to biodiversity, particularly through their interactions with other drivers of change (MEA, 2005; Vila *et al*., 2011). There is an urgent need to anticipate which IAS are likely to cause future problems so that preventative action can be taken promptly.

There are a number of international agreements which recognize the negative effects of IAS and reflect widespread concerns. For example, European countries must ‘strictly control the introduction of nonindigenous species’ (Bern Convention on the Conservation of European Wildlife & Natural Habitats, 1979, http://conventions.coe.int/Treaty/EN/Treaties/Html/104.htm). The Convention on Biological Diversity (CBD) advocates a three-stage hierarchical approach to IAS: prevention; early detection and rapid eradication; containment and long-term control containment (http://www.cbd.int/invasive/background.shtml). Prevention is the most cost effective and environmentally desirable of these three and is therefore seen as a priority by the CBD. If an IAS has already been introduced then early detection and management are crucial to prevent establishment. Both of these measures can be informed by determining the alien species that are most likely to invade new territories (Shine *et al*., 2010).

Horizon scanning is defined as a systematic examination of potential threats and opportunities within a given context. Horizon scanning to prioritize the threat posed by potentially new IAS which are not yet established within a region is seen as an essential component of IAS management (Copp *et al*., 2007; Shine *et al*., 2010). The GB Non-Native Species Information Portal (GB-NNSIP) was developed to provide information to underpin research and policy for the management of IAS within Great Britain (Roy *et al*., 2014). Horizon scanning is one component of the GB-NNSIP. Horizon scanning has gained a high profile through the publication of lists such as the ‘100 of the World's Worst IAS’ (compiled by the Global Invasive Species Database – http://www.issg.org/database/species/search.asp?st=100ss) and the DAISIE (Delivering Alien Species Inventories for Europe) ‘100 of the Worst’ (http://www.europe-aliens.org/speciesTheWorst.do). In addition, a number of EU frameworks have implemented horizon scanning across selected sectors such as plant and animal health (Shine *et al*., 2010). Horizon scanning has historically included extensive literature reviews, to ascertain species of concern and generally (but not always) some form of risk assessment (Essl *et al*., 2011). However, the importance of risk assessment tools is increasingly recognized as a component of approaches to identify potential future IAS not already present within a region (Essl *et al*., 2011). Risk assessment tools based on a specified set of criteria have been developed for a number of countries (Randall *et al*., 2008; Branquart, 2007; Essl *et al*., 2011) including Great Britain (Booy *et al*., 2006; Copp *et al*., 2009). In general, these are employed for prioritizing alien species already present according to their impact (Randall *et al*., 2008) although their potential for identifying future IAS that are not already present is recognized (Essl *et al*., 2011).

There have been a number of horizon-scanning exercises for IAS in Great Britain but these have involved discrete taxonomic groups, such as plants (Thomas, 2010b) or animals (Parrott *et al*., 2009), or distinct environments such as freshwater (Gallardo & Aldridge, 2013a). In addition, these previous approaches to horizon scanning have not been consensual; they have relied on information from the literature coupled with risk assessment frameworks or modelling approaches. Where small groups of experts have been involved, the final ranking was based on amalgamating scores, so assuming that expert-derived scores are accurate and consistent across species and environments. Here, we describe a method for horizon scanning that combines the structured approaches of literature review and risk assessment (Branquart, 2007) with dynamic consensus methods (Sutherland *et al*., 2011). Our geographical focus was Great Britain but the methods are applicable to other countries or regions. We report on the species derived from this horizon-scanning approach, including information relevant to the invasion process which could be used for underpinning and prioritizing management for both the species and, perhaps more importantly, their pathways of arrival.

Our aim was to create an ordered list of IAS (all plant and animal taxa, excluding microorganisms, across environments) that are likely to arrive, establish and have an impact on native biodiversity within the next 10 years. We adopted consensus methods previously used for collaboratively identifying priorities in various ecological contexts (Sutherland *et al*., 2011) with novel modifications to address this specific question. Consensus methods are in part underpinned by the so-called ‘wisdom of the crowd’ (Galton, 1907; Lorenz *et al*., 2011) in which the aggregate of many people's estimates tends to be closer to the true value than all of the separate individual guesses. However, consensus methods involving experts could be more appropriately described as using ‘wisdom from the crowd’ (Sutherland & Woodroof, 2009). Alongside, systematic methods for gathering and reviewing information (literature reviews and risk assessments), consensus methods provide a robust and repeatable means of collaborative decision-making leading to prioritization.

As our aim was to scan the horizon, we focused on species that had not yet become established, that is had not formed self-sustaining populations (Blackburn *et al*., 2011) in Great Britain in the wild. However, a few species were included which had formed transient local populations that had been detected and had either failed to persist or had been deliberately removed. In accordance with definitions outlined by the CBD (http://www.cbd.int/invasive/background.shtml), we categorized species as alien if their arrival was likely to be mediated by human activities; species that were deemed likely to arrive by natural dispersal from their native range were excluded from consideration. To ensure the scope of the study was achievable, we further confined our attention to species that were likely to impact on biodiversity. We did not consider potential economic, social, or human health impacts, although it should be noted that some of the species that we selected may have such impacts in addition to their effects on biodiversity.

## Materials and methods

**Figure 1:**
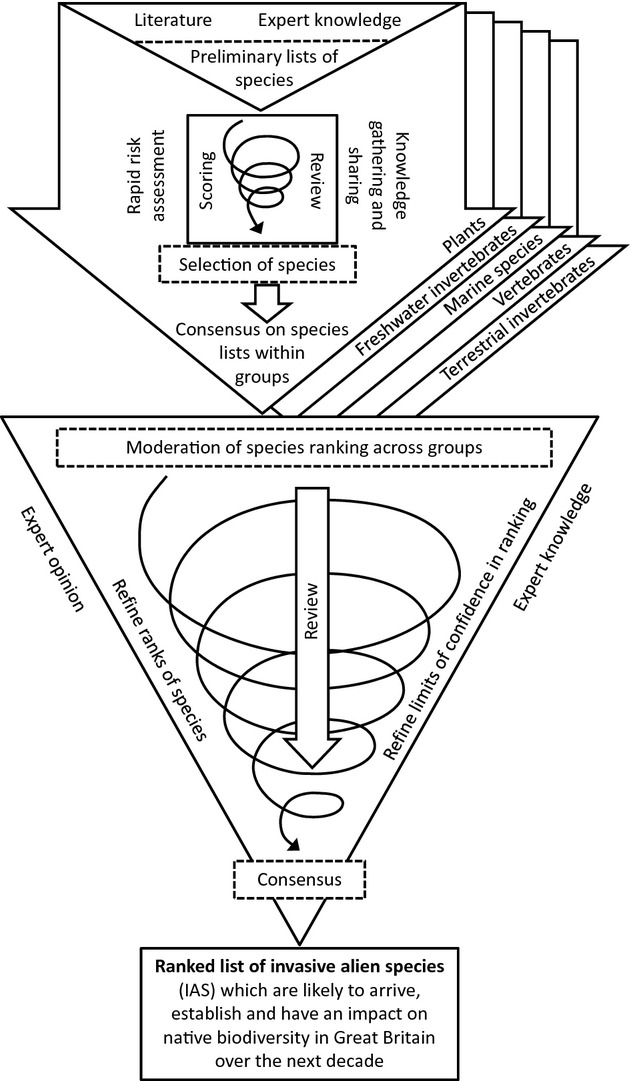
Horizon-scanning process, based on consensus method (Sutherland *et al*., 2011), to derive a ranked list of IAS which are likely to arrive, establish and have an impact on native biodiversity in Great Britain over the next decade. The process involved two distinct phases: preliminary consultation between experts within five expert groups (upper arrows) and consensus-building across expert groups (lower triangle).

We used an adapted version of the consensus method (Sutherland *et al*., 2011) to derive a ranked list of IAS. The process involved two distinct phases (Fig.1):

1. Preliminary consultation between experts within five expert groups (plants, terrestrial invertebrates, freshwater invertebrates, vertebrates and marine species).
2. Consensus-building across expert groups.

### Preliminary consultation

Each of the five expert groups (plants, terrestrial invertebrates, freshwater invertebrates, vertebrates and marine species) comprised two leaders (scientists with relevant ecological and invasion biology expertise) and three to five additional participants selected by the group leaders based on their relevant experience in this field. Twenty-eight participants with complementary expertise across taxonomic groups and environments were chosen to ensure groups had sufficient collective knowledge.

Each expert group was given the task of collating a list of alien species, relevant to their specific group, that are likely to arrive within the next decade, establish and impact on native biodiversity, together with supporting evidence (generally peer-reviewed publications but also grey literature where the former was lacking). Participants were provided with relevant reference sources (Parrott *et al*., 2008, 2009; DAISIE, 2009; Thomas, 2010a) but were also instructed to review and supplement the lists using other literature sources and their own and others' expert opinion.

Each expert group was provided with a spreadsheet template to ensure consistency in the collated information. The grid had the following headings: species, taxonomic group, functional group, native range, likely pathway of arrival, comments and references. Guidance notes were provided on completing the grid. Functional groups were classified as primary producer, herbivore, omnivore, predator and parasite. Pathways of arrival were defined following the classification outlined by Hulme (2009). Each group standardized the assessment of the threat by scoring each of the likelihood of arrival, likelihood of establishment and likelihood of impact on biodiversity from 1 (very unlikely) to 5 (very likely). Impact on biodiversity was assessed by considering the following parameters adapted from Branquart (2007):

1. Dispersal potential
2. Colonization of high conservation value habitats
3. Adverse impacts on native species:
  - Predation/herbivory
  - Competition
  - Transmission of pathogens and parasites to native species
  - Genetic effects
4. Alteration of ecosystem functions:
  - Modification to nutrient cycling
  - Physical modifications to the habitat
  - Modifications of natural successions
  - Disruption of food webs

An overall score for each species was determined as the product of the scores for likelihood of arrival, establishment and impact (maximum score = 125). The overall scores were used to rank the species within the expert groups into categories of low, medium and high risk in preparation for the next phase of the exercise. Participants reviewed and amended scores of the alien species within their group to produce an agreed ranked list of species within each group. This preliminary consultation phase [combining elements of literature review, rapid risk assessment and consensus methods (within groups)] was conducted over 3 months. All group participants were given an opportunity to contribute expertise through e-mail and telephone discussions. These discussions, representing a collaborative approach to decision-making, continued until consensus within the group was achieved. The scores were only used to provide guidance for ranking the species, enabling a starting point from which experts, across groups, could engage in debate leading to modification of the score in some cases. For transparency, we retained the original scores. Only species considered to have an medium or high probability (scores of 3 or above) in all categories (arrival, establishment and impact) were taken forward to the next phase of the process (consensus-building across expert groups); hence the resultant initial lists varied in length across groups from 27 to 74 species (Table1).

**Table 1:** Number of species within each expert group considered at each stage of the horizon-scanning process: preliminary consultation, consensus-building and list of top 30 potential IAS

Expert group	Number of species considered during preliminary consultation	Number of species considered during consensus-building	Number of species within top 30
Plants	113	74	4
Freshwater invertebrates	41	32	5
Marine species	59	52	8
Vertebrates	335	60	7
Terrestrial invertebrates	43	27	6
Total	591	245	30

### Consensus-building across expert groups

Consensus-building across the expert groups took place at a workshop held at the Centre for Ecology & Hydrology (Wallingford, Oxfordshire, UK) over 2 days (25 and 26 April 2013). The group leaders attended on the first day and provided an overview of the species within their lists with particular emphasis on justification of scores. The aim of this exercise was both to review the lists and to ensure standardization of approach to the overall scores derived within groups through the preliminary consultation. Subsequent discussions between group leaders enabled the moderation of group scores, to create an aggregated, ranked list of species from all groups. The list of IAS was then reviewed and expert opinion was used further to refine the ranking. The processes of collaborative review and consensus-building were repeated until the entire group had converged on a ranked list. Throughout the discussions, the group provided expert opinion to support the decision-making process and the scores were used only as guidance for this process. Discussions were further informed by information on uncertainty, usually a consequence of lack of available information, although this was not formalized as an additional metric. Representatives from the Department for the Environment, Food and Rural Affairs (Defra) and the Non-Native Species Secretariat were invited to observe the process and contribute to methodological discussion.

On the second day, group leaders were joined by all expert group participants. The day began with a plenary session in which group leaders provided an overview of the species within their list and the moderated scores. Participants then divided into their expert groups to discuss and further refine the scores of the species within their lists. There was also an opportunity to include additional species if they had been overlooked, or remove species based on the latest information (e.g. to exclude species that have recently become established). The discussions enabled participants to review available information and consider uncertainty in preparation for the final session.

All participants reconvened for 2 h to review and refine the compiled and ranked multi taxon, cross-habitat list of alien species. Ultimately, consensus was reached on the basis of expert opinion provided through open discussion (a transparent process in which questions were openly asked and defences were given or opinions were modified) and majority voting. Discussions were most detailed for species ranked as high impact (with a high degree of certainty) within the aggregated list. For these species, extensive consideration was given to the biology of the species and to life history traits such as dispersal, capacity to survive current and projected future climatic conditions in Great Britain, and known impacts on biodiversity within other invaded countries.

The initial intention was to rank all species numerically, but the workshop group quickly reached consensus that this was unachievable and not necessarily desirable. Instead, the group agreed that grouping species into ranked categories was a preferable approach. In this session, the group decided by iteration how many species should be contained in each of these ranked groups. The species allocated the top position on the list was agreed unanimously by participants from all groups. Participants were requested to reach consensus on the species ranked in positions 2–10, 11–20 and 21–30 but without ascribing any order to species within these groups. The remaining species were categorized as medium risk. Low risk species (those which scored less than three in one or more of the arrival, establishment and impact categories) had been removed in the previous (preliminary consultation) stage.

The expert groups were given a few weeks following the meeting for extraction of further information from the literature to fill gaps on various attributes of the species within the top 30 (such as pathways of arrival, comments on invasion biology and key references), although none of this extra information necessitated any revision of the likelihood scores or consensually agreed rankings. The original scores derived through the consultation process are reported (Table2). However, the scores were only used as a guide and the outcomes of the discussions between experts overruled the scores leading to the final ranked list.

**Table 2:** The highest-risk future alien invasive species in Great Britain (based on their likelihood of arrival, establishment and impact on native biodiversity over the next 10 years) derived from consensus-building among experts

Rank	Species	Common name	Taxonomic Group	Functional Group	Environment	Native range	Pathway of arrival	A	B	C	Overall score (A × B × C)
1	*Dreissena rostriformis bugensis*	Quagga mussel	Mollusca: Bivalvia	Omnivore	F	Ponto-Caspian	SA	5	5	5	125
2–10	*Anoplophora glabripennis*	Asian longhorn beetle	Insecta: Coleoptera: Cerambycidae	Herbivore	T	China	For, Nat	5	4	5	100
2–10	*Hemigrapsus sanguineus*	Asian shore crab	Crustacea: Brachyura	Predator	M	Asia (Pacific)	SA	5	5	4	100
2–10	*Hemigrapsus takanoi*	Brush-clawed shore crab	Crustacea: Brachyura	Predator	M	Asia (Pacific)	SA	5	5	4	100
2–10	*Homarus americanus*	American lobster	Crustacea: Astacidea	Predator	M	North America	Aq	5	4	5	100
2–10	*Myriophyllum heterophyllum*	American water-milfoil	Angiosperm: Haloragaceae	Primary producer	F	North America	Orn	5	5	4	100
2–10	*Neogobius melanostomus*	Round goby	Perciformes: Gobiidae	Predator	F	Ponto-Caspian	SA	4	5	5	100
2–10	*Procyon lotor*	Raccoon	Mammalia: Carnivora	Predator	T	North and CentralAmerica	Orn	5	4	5	100
2–10	*Threskiornis aethiopicus*	African sacred ibis	Aves: Pelecaniformes	Predator	T	Sub-Saharan Africa	Nat	5	4	5	100
2–10	*Vespa velutina*	Asian hornet	Insecta: Hymenoptera: Vespidae	Predator	T	China	SA, P, Nat	5	5	4	100
11–20	*Thaumetopoea pityocampa*	Pine processionary moth	Insecta: Lepidoptera: Thaumetopoeidae	Herbivore	T	Mediterranean region, North Africa, MiddleEast	For, Nat	5	4	5	100
11–20	*Baccharis halimifolia*	Sea myrtle, saltbush	Angiosperm: Asteraceae	Primary producer	T	North America	Orn	5	5	4	100
11–20	*Corbicula fluminalis*	Asian clam	Mollusca: Bivalvia: Corbiculidae	Omnivore	F	Eastern Asia	SA	4	5	5	100
11–20	*Corvus splendens*	Indian house crow	Aves: Passeriformes	Omnivore	T	Southern Asia	SA	4	4	5	80
11–20	*Echinogammarus trichiatus*	Curly haired urchin shrimp	Crustacea: Gammaridea	Omnivore	F	Ponto-Caspian	SA	5	5	3	75
11–20	*Linepithema humile*	Argentine ant	Insecta: Hymenoptera: Formicidae	Predator	T	South America	SA, P, Nat	5	3	5	75
11–20	*Mnemiopsis leidyi*	American comb jelly	Ctenophora: Lobata	Predator	M	North America and South America	SA	5	5	4	100
11–20	*Nassella neesiana (Stipa neesiana)*	Chilean needle grass	Angiosperm: Poaceae	Primary producer	T	South America	Orn	5	5	3	75
11–20	*Proterorhinus marmoratus*	Tubenose goby	Actinopterygii: Perciformes	Predator	F	Ponto-Caspian	SA	4	5	5	100
11–20	*Rapana venosa*	Veined rapa whelk	Mollusca: Gastropoda	Predator	M	Asia (Pacific)	SA, Aq	5	4	5	100
21–30	*Agrilus plannipennis*	Emerald ash borer	Insecta: Coleoptera: Buprestidae	Herbivore	T	Asia	For, Nat	3	5	4	60
21–30	*Celtodoryx ciocalyptoides*	A sponge	Porifera: Poecilosclerida	Omnivore	M	Pacific	SA, Aq	5	4	3	60
21–30	*Dryocosmus kuriphilus*	Oriental chestnut gall wasp	Insecta: Hymenoptera: Cynipidae	Herbivore	T	China	For, Nat	3	5	3	45
21–30	*Echinogammarus ischnus*	Bald urchin shrimp	Crustacea: Gammaridea	Omnivore	F	Ponto-Caspian	SA	5	5	3	75
21–30	*Gyrodactylus salaris*	Salmon fluke	Platyhelminthes: Trematoda	Parasite	F	Baltic	HF, Aq, SA	5	4	4	80
21–30	*Microstegium vimineum*	Japanese stiltgrass	Angiosperm: Poaceae	Primary producer	T	Central and eastern Asia	**SC**, RM	3	4	5	60
21–30	*Nyctereutes procyonoides*	raccoon dog	Mammalia: Carnivora	Predator	T	Eastern Asia (Vietnam to Russia)	Orn	4	3	5	60
21–30	*Ocenebra inornata*	Japanese sting winkle	Mollusca: Gastropoda	Predator	M	Asia (Pacific)	SA, Aq	5	4	4	80
21–30	*Tamias sibiricus*	Siberian chipmunk	Mammalia: Rodentia	Omnivore	T	Northern Asia (Kazahkstan to Japan)	Orn	5	4	4	80
21–30	*Gracilaria vermiculophylla*	Rough agar weed	Rhodophyta: Gracilariaceae	Producer	M	Pacific	SA, Aq	5	5	4	100

*Dreissena rostriformis bugensis* was unanimously considered to be the highest ranking species. The others are ranked equally within categories of 2–10, 11–20 and 21–30. Functional groups are provided alongside environment (F = freshwater, M = marine, T = terrestrial), native range and pathway of arrival [For = forestry (species introduced to benefit forestry), Aq = aquaculture (species introduced into aquatic environments for use by humans but excluding ornamental species), Orn = ornamental (species introduced as garden plants, zoo animals and pets), HF = hunting/fishing (species introduced for recreational hunting and fishing), P = produce (species arriving on imported food or flowers), SC = seed contaminant (species arriving on seeds), RM = raw material (species arriving on raw materials such as timber), SA = stowaway (species arriving through transport such as boats, aircraft and land vehicles) and Nat = natural spread (species arriving through colonization from previously invaded regions)]. Scores of 1 (very unlikely) to 5 (very likely) were given for likelihood of arrival (A), likelihood of establishment (B) and likelihood of impact (C). The overall score (A × B × C) was used for preliminary ranking of all species, but the final ranking was achieved by consensus-building discussion.

## Results

**Figure 2:**
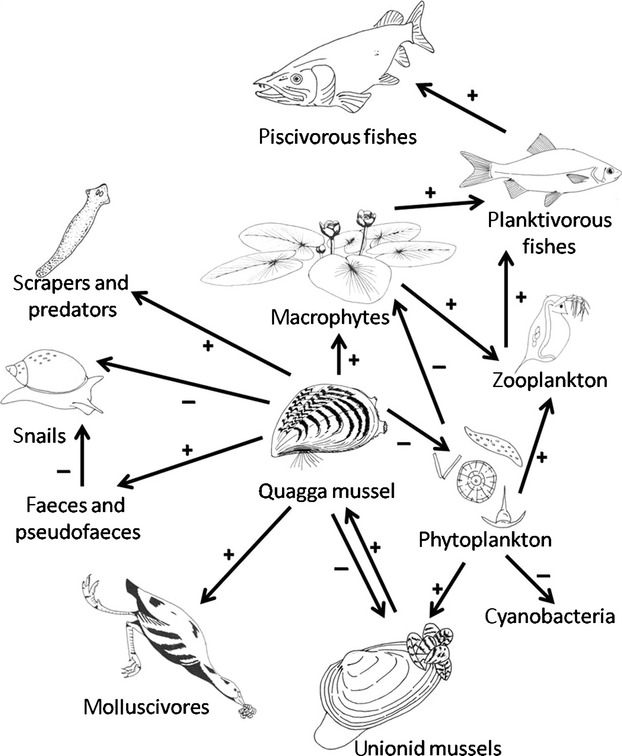
Possible major direct and indirect effects of quagga mussels on Britain's freshwater ecosystems. Details of interactions are provided in the text. Beneficial effects are indicated with a plus sign, negative effects with a minus sign. Figure adapted and revised from MacIsaac (1996).

Ninety-three species not native to Great Britain were agreed to constitute at least a medium risk (based on score and consensus) with respect to arriving, establishing and posing a threat to native biodiversity (Tables2, S1 and S2). The quagga mussel, *Dreissena rostriformis bugensis*, received maximum scores for all three criteria and was unanimously ranked in the top position (Table2; Fig.2). A further 29 species were ranked as being of high risk. It was agreed by consensus that it was inappropriate to individually rank these species, but that they could be placed in ranked classes (positions 2–10, then 11–20 and 21–30) with decreasing levels of risk (Table2). The remaining 63 species were considered to be medium risk, and are presented in an unranked long list (Table S2).

**Figure 3:**
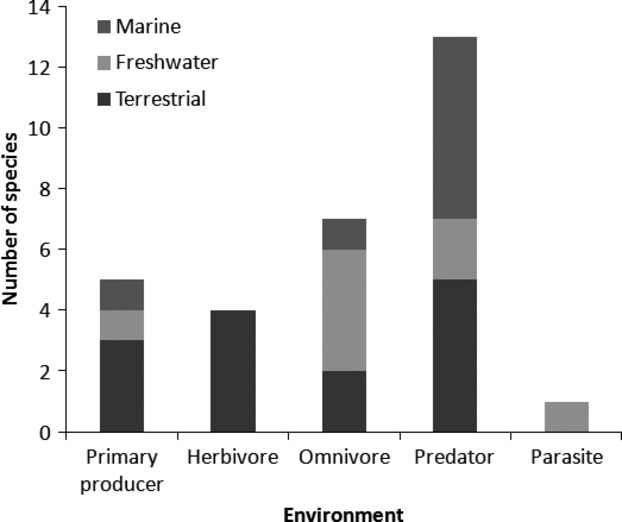
Number of species ranked within the top 30 potential IAS within different functional groups (primary producer, herbivore, omnivore, predator and parasite) predicted to arrive into different environments (terrestrial, freshwater and marine) in Britain.

The top 30 species included representatives from terrestrial, freshwater and marine environments and across a range of functional groups (Table2; Fig.3). Most species were categorized as terrestrial (14 species) followed by marine (8 species) and freshwater (8 species). Predatory species dominated the list (13 species). Within freshwater environments, omnivorous species were the most numerous, closely followed by predatory species. In contrast, predatory species were most numerous in terrestrial environments (five species) with herbivorous species the next most numerous (four species). Only one parasitic species, *Gyrodactylus salaris* (salmon fluke), was ranked within the top 30 (although several were excluded from consideration because their impact was on economically significant, nonnative species).

**Figure 4:**
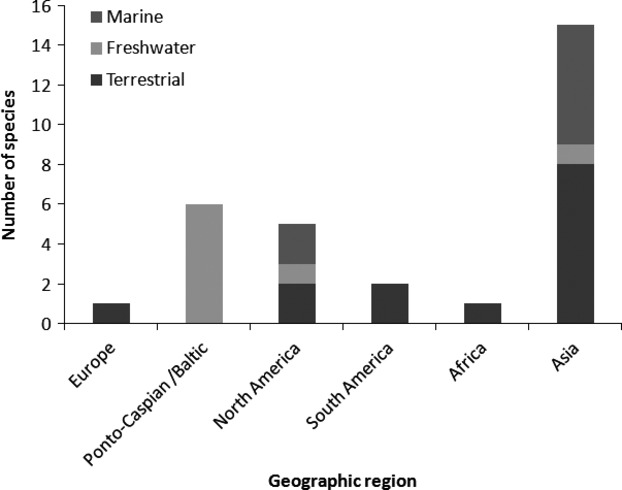
Number of species ranked within the top 30 potential IAS predicted to arrive in Britain into different environments (Terrestrial, Freshwater and Marine) from different geographic regions (Europe, Ponto-Caspian and Baltic Seas, Asia, North America, South America and Africa).

**Figure 5:**
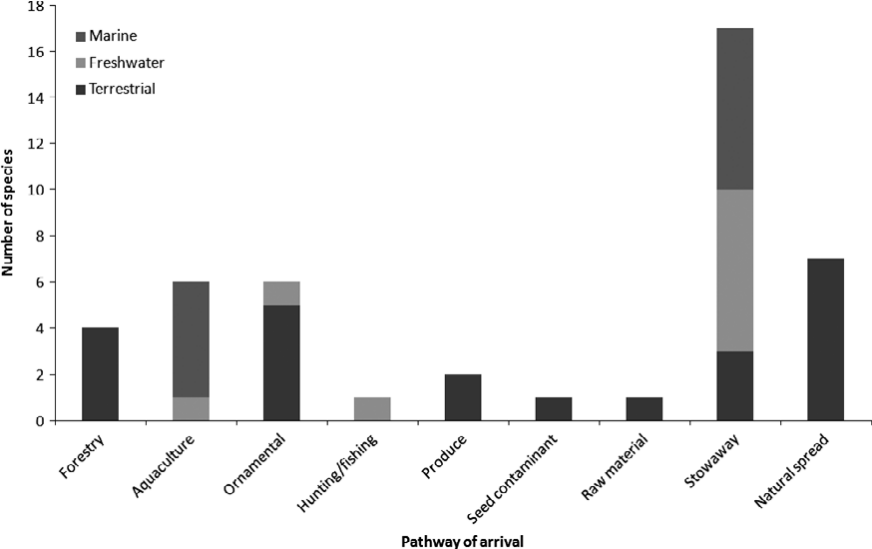
Number of species ranked within the top 30 potential IAS predicted to arrive in Britain by different pathways (defined within Table2) into different environments (Terrestrial, Freshwater and Marine).

Most of the terrestrial and marine species within the list of 30 species originate from Asia (Table2; Fig.4). In contrast, most new freshwater arrivals are predicted to originate from the Ponto-Caspian region. The stowaway pathway (in land, air or sea transport vehicles) is likely to be the most common mechanism of introduction (Table2; Fig.5). However, the species listed span a range of pathways and multiple pathways of introduction are anticipated for many of the species.

## Discussion

We found that a consensus approach to horizon scanning, which combines available evidence and expert opinion, was a practical way to derive a list of alien species that have a relatively high probability of arrival, establishment and becoming invasive (spread and impact on native biodiversity). Comprehensive horizon scanning requires breadth of expertise across taxonomic groups and environments. Convening participants with complementary expertise ensures sufficient collective knowledge to undertake the process of identifying and ranking relevant alien species in an open, rigorous and time-efficient way. Despite initial doubts that agreement would be reached among such a heterogeneous group of experts, there were surprisingly few concerns raised from the participants throughout this consensus approach to horizon scanning though there was robust debate. We found that it was essential to clearly define relevant terms (‘establishment’ and ‘alien species’) and to define the remit of the exercise explicitly (i.e. only considering alien species with the potential to impact on native biodiversity, and excluding any consideration of other impacts) to ensure consistency across expert groups. Given this clear remit, we found that consensus across all participants was achieved, both on the ranking of species and on modifications to the method during the process (e.g. the decision to rank species in broad risk groups, and the number of species in each group). This suggests that, although our approach was modified from that described by Sutherland *et al*. (2011), this highly focussed consensus-building is a potentially effective way to prioritize even across individuals with diverse expertise considering a breadth of taxa and environments. Great Britain was the focus of our study but the methods are applicable in different regions across the world.

Of course the accuracy of the predictions resulting from this approach will only be tested over time. By its nature, horizon scanning uses expert opinion to extrapolate from an incomplete evidence base. Many uncertainties inevitably remain at the conclusion of such an exercise, even for species whose invasion and impact history elsewhere has been well documented. It is impossible to be certain how species will respond, when placed into the unprecedented context of a different climate and complex novel interactions with other species. For this reason, we did not attempt to quantify the uncertainty associated with the impact of each IAS on native biodiversity, preferring instead to integrate this consideration into the iterative discussions within and across the expert groups.

The species in the top 30 include representatives from a range of functional groups and environments, and with native distributions across a range of biogeographic regions. It is intuitive that species already present in locations close to Great Britain are more likely to have already arrived in Great Britain than those from greater distances because of a long history of close transport and trade links (Preston *et al*., 2004; Baker & Hills, 2008). Hence, it is not surprising that the rate of IAS arriving from continental Europe to Great Britain is slowing while, in striking contrast, there is a dramatic increase in the rate of new arrivals from temperate Asia (Roy *et al*., 2012). At least 35 Ponto-Caspian species have spread into Western Europe over the past three decades as a result of extensive canal construction, increasing the interconnectivity of waterways between these two regions (Bij De Vaate *et al*., 2002). Likewise, Britain's freshwaters have received species of Ponto-Caspian origin at increasing rates (Keller *et al*., 2006; Gallardo & Aldridge, 2013c). This is likely to continue, and we expect that further immigrants from this area will include our top-ranked species, the quagga mussel.

The quagga mussel is a dreissenid bivalve mollusc native to the Ponto-Caspian region of Eastern Europe to which we gave maximum scores for risk of arrival, establishment and impact. This species is now well established in the Netherlands, a country that has strong bioclimatic similarity with much of England (Gallardo & Aldridge, 2013c) and has considerable trade exchange with Britain (Talbot *et al*., 2009). The species is readily transported in ballast water and overland in association with recreational boat traffic (Sylvester & MacIsaac, 2010), making its arrival extremely likely. The severity of the impact of the quagga mussel relates to its function as an ecosystem engineer: it can become the dominant benthic organism within invaded systems (Sousa *et al*., 2009) with a wide range of direct and indirect impacts (Fig.2). Clearer water, resulting from the filtering capacity of the quagga mussel (Cross *et al*., 2010), can lead to changes in the diversity and abundance of phytoplankton and zooplankton communities. This in turn can result in the competitive release of cyanobacteria, thus causing toxic blooms (MacIsaac, 1996). It also benefits bottom-rooting macrophytes which can become more abundant in the presence of quagga mussels (Aldridge *et al*., 2004). Deposition of faeces diverts nutrients to the benthos and alters sediment structure causing an increased density of scrapers and predators (especially leeches, flatworms and mayflies), but reduces abundance of large snails, sphaeriid clams and burrowing amphipods (Ward & Ricciardi, 2007; Sousa *et al*., 2009). Fouling by quagga mussels can have impacts on unionid mussels (Schloesser *et al*., 2006), potentially including species of conservation concern (Sousa *et al*., 2011). Bioclimatic models predict a 75% overlap in the fundamental niche of zebra and quagga mussels, suggesting the niches of the two species are similar but nonetheless significantly different (Quinn *et al*., 2013). Therefore, zebra mussels are already widely established across Great Britain should not lead to complacency about the invasion of quagga mussels.

Ecosystem engineers, such as the quagga mussel, and other species with the potential to disrupt community structure are likely to exert considerable pressure on native biodiversity. Therefore, it is not surprising that the top 30 IAS are dominated by species that could have these effects. For example, the Asian hornet, *Vespa velutina*, is a major predator of social insects, especially honeybees, but also other invertebrates, with important potential consequences for native biodiversity and pollination services (Villemant *et al*., 2011). The Asian hornet was first reported in south-west France in 2004, probably having been accidentally imported from its native Asian range through the horticultural trade. It is now considered to be established in France and is spreading rapidly north and east. Niche modelling indicates that Britain is climatically suitable for this species (Villemant *et al*., 2011). The two predatory gastropods included within the top 30, *Rapana venosa* and *Ocenebra inornata*, have the potential to consume bivalves in large numbers and so ultimately adversely affect the formation of reefs (Micu & Todorova, 2007).

Terrestrial vertebrates are responsible for the greatest range of impacts (Vilà *et al*., 2010). Five terrestrial vertebrates are included within the list of species ranked within the top 30. Sacred ibis (*Threskiornis aethiopicus*) is a large colonial wading bird originating in Africa with established nonnative breeding populations in France. It is a species that is commonly kept in bird collections in the UK (Baker & Hills, 2008). Largely carnivorous opportunistic feeders, sacred ibis are known to prey heavily on populations of birds, amphibians, fish and invertebrates and as such have the potential for major impacts on biodiversity. The raccoon, *Procyon lotor*, is a highly adaptable omnivorous mammal originating from North America, which as a result of escapes and deliberate introductions in the mid-20th century has established two large populations in Germany (numbering 200 000–400 000 individuals) as well as populations in France, Belarus and Azerbaijan. Raccoons, which are kept as pets in Britain, periodically escape or are released, and have the potential to establish populations and effect biodiversity through predation and disease transmission (Bradley & Altizer, 2006; Baker & Hills, 2008; Beltran-Beck *et al*., 2012).

The transmission of pathogens from alien species to native species, through the process of spillover (Roy & Lawson-Handley, 2012), represents a considerable threat to biodiversity. A number of species ranked within the top 30 have the potential to transmit disease including raccoons and, for example, the American lobster, *Homarus americanus*. The American lobster has the potential to interbreed with the native lobster *Homarus gammarus* but it is also known to carry gaffkaemia, a bacterial disease, known to be lethal to *H. gammarus* (Stebbing *et al*., 2012).

The multidisciplinary nature of the exercise raised some challenges that were not fully resolved through the process but are worth considering. Cryptic species, which are difficult to distinguish from one another using morphological characteristics, were raised as a problem for some taxonomic groups, especially marine animals and plants, and are known to present a particular challenge to understanding biological invasions (Avery *et al*., 2013). Cryptic species are a problem for three reasons. Firstly, confusion about species limits or species nomenclature causes problems for IAS legislation (and hence response) which rely on species definitions. For example, Asian clams (*Corbicula* spp.) have poorly resolved taxonomy (Pigneur *et al*., 2001). Of the three morphotypes in Europe, one, *Corbicula fluminea*, is already present in Great Britain (Aldridge & Muller, 2001) while another is listed in our top 20 under the name by which it is typically referred to, *Corbicula fluminalis* (Bodis *et al*., 2011), but others may be relevant. Secondly, cryptic species make early detection of new species difficult. For example the alien aquatic fern, *Azolla filiculoides*, is well established in Britain, but the morphological characters distinguishing it from *Azolla caroliniana* are unclear so it is not known whether both species are actually present. Similarly, *Heracleum mantegazzianum* is established in Britain but may be confused with similar-looking species which are known to be spreading in Northern Europe including *Heracleum persicum* and *Heracleum sosnowskyi*. Thirdly, the extent to which cryptic species represent a threat to native biodiversity is often uncertain, particularly because potential impacts may have already been made by a congeneric species or subtly different niche requirements of the various cryptic species may lead to additional impacts. In addition, taxa classified below the species level (subspecies and karyotypes) may also be considered invasive and would certainly raise further difficulties when horizon scanning, but were not considered in our species-level approach. It is sometimes difficult to establish whether or not a species should be classified as alien. For example, a particular problem for marine species is that it can be very difficult to distinguish between natural dispersal of a species from its native range and movement by human agency and, therefore, categorization as alien. Establishing actual or most likely pathways of arrival for IAS can be challenging, even retrospectively. Some of the species could arrive via more than one pathway, making it difficult to assess the likelihood of arrival (Hulme, 2009).

Overall, we found that knowledge gaps for terrestrial invertebrates were far greater than for other taxonomic groups (Kenis *et al*., 2009). In particular, the paucity of ecological information on many species constrained our ability to derive comprehensive lists of species for ranking (Roy *et al*., 2011a,b). For example, even well-studied groups such as Lepidoptera, Coleoptera and social Hymenoptera contained many potentially problematic species for which information was insufficient to form an evidence-based judgement. For the same reason, our list lacks any insect parasitoid species even though their impact on biodiversity could be far-reaching (Henneman & Memmott, 2001). Lack of information does not denote absence of threat. Nevertheless, we took a deliberately conservative approach, including in our list only those species with good supporting evidence of impacts on biodiversity. We note, however, that our wide-ranging discussions on many species with limited published evidence of impacts on biodiversity did not result in any additional species being included within the top 30.

For most terrestrial invertebrates, research on impacts is focussed on commercial interests, such as forestry, or human health and well-being, rather than impacts on biodiversity (De Clercq *et al*., 2011; Roy *et al*., 2011a). Indeed all six terrestrial invertebrates listed in our top 30 have known impacts either on forestry or as nuisance species, and have received much more attention for these than for their impact on biodiversity.

It was recognized that assessment of vascular plants also posed some difficulties because the process of invasion tends to be very slow when compared to mobile vertebrates and invertebrates. There is often a time lag between species arriving through the horticultural trade and establishing in the wild where they may impact on biodiversity. Furthermore, many of the potentially threatening species have already ‘arrived’ in Great Britain through ornamental and horticultural pathways; although species currently grown in gardens or in planting schemes in urban habitats were not included within our assessment. Escapees from this pool of species are more likely to become established than new arrivals because of their propensity to grow and reproduce in Great Britain. We recommend that any future horizon scanning for invasive alien plants should include casual species (i.e. which are not self-sustaining but have high potential for establishment) and those in cultivation outdoors in gardens. Such an approach would be comparable to assessments completed recently for other European countries (Veerlove, 2006; Pyšek *et al*., 2012). This could be reinforced by an assessment of plant species that have become categorized as IAS in similar eco-climatic ranges. An analogous situation exists for some other taxa, for example, the many species of waterfowl already kept in captivity in Great Britain.

It is important to note the unpredictable nature of IAS introduction events and, therefore, recognize the imperfect nature of horizon-scanning lists. Horizon scanning is only one component of the three-stage hierarchical approach proposed by the CBD for managing the impacts of IAS. Communication and cross-boundary collaborations, ensuring knowledge on IAS is shared between countries, are essential to ensure successful implementation of IAS strategy. There tends to be a time lag between a species arriving in mainland Europe and subsequent spread to Great Britain (Gallardo & Aldridge, 2013b) and as such Great Britain is more likely to benefit from early warning from neighbouring countries. Indeed, the recent arrival of the Asian hornet in France was not anticipated, but effective and rapid communication has been an important component of the early warning process, ensuring that other countries are prepared for the arrival of this IAS. The GB Non-Native Species Secretariat has co-ordinated contingency plans in relation to the Asian hornet following notification of its arrival in France. Horizon scanning is extremely difficult in regions where neighbouring countries have not collated information on IAS or for countries that have climatic or geographic conditions that increase the probability of them being subject to the first record of an IAS.

There are a number of ways in which this research could be extended. We did not consider the biodiversity impacts of measures taken to manage IAS once established, even though control strategies directed at certain IAS are known to have had knock-on consequences for native biodiversity (Heimpel *et al*., 2010). Consideration of the effects of strategies employed to manage IAS after establishment would enhance understanding of the far-reaching consequences of invasion by some species. We also did not take into account differences among candidate species in the effectiveness of any control measures needed to eradicate them. While it is clear that some new IAS would be harder to deal with than others, our aim was simply to rank species on their risk of arrival, establishment and impact on biodiversity. It is clear that IAS vary substantially in their impacts. Hence, it is important to prioritize IAS which poses immediate and significant threats. Perhaps more importantly, the pathways through which they arrive into a country should also be prioritized. The intention of this paper is to provide a basis for highlighting those IAS that may pose the greatest risk to biodiversity in Great Britain over the next decade, but the methods have global applicability.

## Supporting Information

**Table S1.** The highest-risk future alien invasive species in Great Britain (based on their likelihood of arrival, establishment and impact on native biodiversity over the next 10 years) derived from consensus-building among experts. *Dreissena bugensis* was unanimously considered to be the highest ranking species. The others are ranked equally within categories of 2–10, 11–20 and 21–30. Functional groups are provided alongside environment (F = freshwater, M = marine, T = terrestrial), native range and pathway of arrival (For = forestry (species introduced to benefit forestry), Aq = aquaculture (species introduced into aquatic environments for use by humans but excluding ornamental species), Orn = ornamental (species introduced as garden plants, zoo animals and pets), HF = hunting/fishing (species introduced for recreational hunting and fishing), P = produce (species arriving on imported food or flowers), SC = seed contaminant (species arriving on seeds), RM = raw material (species arriving on raw materials such as timber), SA = stowaway (species arriving through transport such as boats, aircraft and land vehicles) and Nat = natural spread (species arriving through colonization from previously invaded regions)). Scores of 1 (very unlikely) to 5 (very likely) were given for likelihood of arrival (A), likelihood of establishment (B) and likelihood of impact (C). The overall score (A × B × C) was used for preliminary ranking of all species, but the final ranking was achieved by consensus-building discussion. Species-specific comments and references are provided.

**Table S2.** Species ranked as posing a medium risk (ranked equally within 31–93) with respect to likelihood of arriving, establishing and having an impact on native biodiversity in Britain over the next 10 years.
