# Supplementary material for: Horizon scanning for invasive alien species with the potential to threaten biodiversity in Great Britain
Source: Glob Chang Biol. 2014 May 19;20(12):3859–71. doi: 10.1111/gcb.12603 (PMC4283593; doi:10.1111/gcb.12603)
Supplement: Supplementary file 1 — Table S1. The highest-risk future alien invasive species in Great Britain (based on their likelihood of arrival, establishment and impact on native biodiversity over the next 10 years) derived from consensus-building among experts. Dreissena bugensis was unanimously considered to be the highest ranking species. The others are ranked equally within categories of 2–10, 11–20 and 21–30. Functional groups are provided alongside environment (F = freshwater, M = marine, T = terrestrial), native range and pathway of arrival (For = forestry (species introduced to benefit forestry), Aq = aquaculture (species introduced into aquatic environments for use by humans but excluding ornamental species), Orn = ornamental (species introduced as garden plants, zoo animals and pets), HF = hunting/fishing (species introduced for recreational hunting and fishing), P = produce (species arriving on imported food or flowers), SC = seed contaminant (species arriving on seeds), RM = raw material (species arriving on raw materials such as timber), SA = stowaway (species arriving through transport such as boats, aircraft and land vehicles) and Nat = natural spread (species arriving through colonization from previously invaded regions)). Scores of 1 (very unlikely) to 5 (very likely) were given for likelihood of arrival (A), likelihood of establishment (B) and likelihood of impact (C). The overall score (A × B × C) was used for preliminary ranking of all species, but the final ranking was achieved by consensus-building discussion. Species-specific comments and references are provided. [file gcb0020-3859-sd1.docx]

**Table S1:** The highest-risk future alien invasive species in Great Britain (based on their likelihood of arrival, establishment and impact on native biodiversity over the next ten years) derived from consensus-building among experts. *Dreissena bugensis* was unanimously considered to be the highest ranking species. The others are ranked equally within categories of 2-10, 11-20 and 21-30. Functional groups are provided alongside environment (F = freshwater, M = marine, T = terrestrial), native range and pathway of arrival (For = forestry (species introduced to benefit forestry), Aq = aquaculture (species introduced into aquatic environments for use by humans but excluding ornamental species), Orn = ornamental (species introduced as garden plants, zoo animals and pets), HF = hunting/fishing (species introduced for recreational hunting and fishing), P = produce (species arriving on imported food or flowers), SC = seed contaminant (species arriving on seeds), RM = raw material (species arriving on raw materials such as timber), SA = stowaway (species arriving through transport such as boats, aircraft and land vehicles) and Nat = natural spread (species arriving through colonisation from previously invaded regions)). Scores of 1 (very unlikely) to 5 (very likely) were given for likelihood of arrival (A), likelihood of establishment (B) and likelihood of impact (C). The overall score (A×B×C) was used for preliminary ranking of all species, but the final ranking was achieved by consensus-building discussion. Species-specific comments and references are provided.

| Rank | Species | Common name | Taxonomic Group | Functional Group | Environment | Native range | Pathway of arrival | A | B | C | Overall score (A×B×C) | Comments | References |
| --- | --- | --- | --- | --- | --- | --- | --- | --- | --- | --- | --- | --- | --- |
| 1 | *Dreissena rostriformis bugensis* | quagga mussel | Mollusca: Bivalvia | Omnivore | F | Ponto-Caspian | **SA** | 5 | 5 | 5 | 125 | Keystone species that attaches in very high densities to hard surfaces in both lentic and lotic systems. Drives ecosystem change through high filtration capacity. Currently displacing zebra mussels in parts of North America and Western Europe, including The Netherlands. | ([Baldwin *et al.*, 2002](#_ENREF_4), [Ward & Ricciardi, 2007](#_ENREF_56)) |
| 2-10 | *Anoplophora glabripennis* | Asian longhorn beetle | Insecta: Coleoptera: Cerambycidae | Herbivore | T | China | **For,** Nat | 5 | 4 | 5 | 100 | Wounding by beetle makes tree liable to secondary pests and pathogenic infection. Recent outbreaks in continental Europe; some recent interceptions in GB, including an outbreak in 2012 in Kent which was controlled. | ([CSL, 2004](#_ENREF_12), [EPPO, 1999](#_ENREF_16)) |
| 2-10 | *Hemigrapsus sanguineus* | Asian shore crab | Crustacea: Brachyura | Predator | M | Asia (Pacific) | SA | 5 | 5 | 4 | 100 | Likely to out-compete and displace native crab species especially the shore crab, *Carcinus maenas*. | ([Dauvin, 2009](#_ENREF_13), [Dauvin *et al.*, 2009](#_ENREF_15), [Jensen *et al.*, 2002](#_ENREF_30)) |
| 2-10 | *Hemigrapsus takanoi* | brush-clawed shore crab | Crustacea: Brachyura | Predator | M | Asia (Pacific) | SA | 5 | 5 | 4 | 100 | Likely to out compete and displace native crab species especially the shore crab, *Carcinus maenas*. | ([Dauvin, 2009](#_ENREF_13), [Dauvin & Delhay, 2010](#_ENREF_14)) |
| 2-10 | *Homarus americanus* | American lobster | Crustacea : Astacidea | Predator | M | North America | Aq | 5 | 4 | 5 | 100 | American lobsters carry a bacterial disease, *Gaffkaemia*, that is lethal to other *Homarus* spp., and an epizootic shell disease may be transferred with American lobsters to Europe. This disease has resulted in the closing of parts of the North American lobster fishery and its impact on European lobster populations/fisheries could be equally severe. Hybridisation with the native *H. gammarus* is also an issue. Continues to be released into GB waters in small numbers following purchase from restaurants, where they are being displayed before cooking, and released into local waters. | ([Jørstad *et al.*, 2011](#_ENREF_32), [Stebbing *et al.*, 2012](#_ENREF_50)) |
| 2-10 | *Myriophyllum heterophyllum* | American water-milfoil | Angiosperm: Haloragaceae | Primary producer | F | North America | **Orn** | 5 | 5 | 4 | 100 | Widely grown in aquaria/ponds and with capacity to become established and highly invasive. | ([EPPO, 2013](#_ENREF_20), [Preston & Croft, 1997](#_ENREF_44), [Thum & Lennon, 2010](#_ENREF_52)) |
| 2-10 | *Neogobius melanostomus* | round goby | Perciformes: Gobiidae | Predator | F | Ponto-Caspian | **SA** | 4 | 5 | 5 | 100 | The numbers of native fish species have declined in areas where the round goby has become abundant. Fish eggs, insect larvae and snails are also eaten. They also show a strong preference for zebra and quagga mussels. | ([Vanderploeg *et al.*, 2002](#_ENREF_54)) |
| 2-10 | *Procyon lotor* | raccoon | Mammalia: Carnivora | Predator | T | North and Central America | Orn | 5 | 4 | 5 | 100 | Regular escape/release from captivity in GB (invariably as single individuals), well established populations in central and western Europe. Potentially major vector of disease, e.g. rabies. | ([Baker & Hills, 2008](#_ENREF_3), [Parrott *et al.*, 2008](#_ENREF_42)) |
| 2-10 | *Threskiornis aethiopicus* | African sacred ibis | Aves: Pelecaniformes | Predator | T | Sub-Saharan Africa | Nat | 5 | 4 | 5 | 100 | Potentially serious predator of birds and amphibians – including species of conservation concern. Well established and spreading in France; individuals seen in GB. | ([Blair *et al.*, 2000](#_ENREF_8), [Parrott *et al.*, 2008](#_ENREF_42), [Yesou & Clergeau, 2006](#_ENREF_59)) |
| 2-10 | *Vespa velutina* | Asian hornet | Insecta: Hymenoptera: Vespidae | Predator | T | China | **SA,** P, Nat | 5 | 5 | 4 | 100 | Important predator, especially of honeybees, other bees & social wasps, and also Diptera. Recent arrival (2004) in S.W. France and spreading rapidly north and east; GB has suitable climate & habitat. | ([EPPO, 2007](#_ENREF_18), [Marris & Roy, 2013](#_ENREF_37), [Villemant *et al.*, 2011](#_ENREF_55)) |
| 11-20 | *Thaumetopoea pityocampa* | Pine processionary moth | Insecta: Lepidoptera: Thaumetopoeidae | Herbivore | T | Mediterranean region, North Africa, Middle East | **For,** Nat | 5 | 4 | 5 | 100 | Economically important pest; urticating hairs pose health hazard; most *Pinus* species susceptible but also certain broadleaved tree species. Abundant in southern & central Europe, expanding northwards in France; transient larval population in GB on imported saplings intercepted in 1995. | ([Baker *et al.*, 2012](#_ENREF_2), [Battisti *et al.*, 2005](#_ENREF_5), [EPPO, 2004](#_ENREF_17)) |
| 11-20 | *Baccharis halimifolia* | sea myrtle, saltbush | Angiosperm: Asteraceae | Primary producer | T | North America | **Orn** | 5 | 5 | 4 | 100 | Forms dense monospecific stands in coastal sites | ([Caño *et al.*, 2013](#_ENREF_11), [EPPO, 2009](#_ENREF_19), [EPPO, 2013](#_ENREF_20), [Etienne *et al.*, 2010](#_ENREF_21)) |
| 11-20 | *Corbicula fluminalis* | Asian clam | Mollusca: Bivalvia: Corbiculidae | Omnivore | F | Eastern Asia | **SA** | 4 | 5 | 5 | 100 | An ecosystem engineer, driving ecological change to river systems through its high densities coupled with large filtration capacity. Widely present across Western Europe, including France and The Netherlands. | ([Bodis *et al.*, 2011](#_ENREF_9)) |
| 11-20 | *Corvus splendens* | Indian house crow | Aves: Passeriformes | Omnivore | T | Southern Asia | SA | 4 | 4 | 5 | 80 | Adaptable and widespread invasive species globally with evidence of negative impact on native birds. Already in close proximity in Netherlands and very likely to arrive in GB by ship. | ([Nyari *et al.*, 2006](#_ENREF_39), [Ottens, 2003](#_ENREF_40), [Ottens & Ryall, 2003](#_ENREF_41)) |
| 11-20 | *Echinogammarus trichiatus* | curly haired urchin shrimp | Crustacea: Gammaridea | Omnivore | F | Ponto-Caspian | **SA** | 5 | 5 | 3 | 75 | Wide diet and high densities suggests a capacity to drive pronounced change to invaded systems. Can be found in lotic and lentic systems, and often associated with rocks, sand and reeds. In The Netherlands the species is often sampled together with zebra mussels. | ([Bij de Vaate *et al.*, 2002](#_ENREF_7)) |
| 11-20 | *Linepithema humile* | Argentine ant | Insecta: Hymenoptera: Formicidae | Predator | T | South America | **SA,** P, Nat | 5 | 3 | 5 | 75 | Strong adverse impacts on native ant species, other arthropods and ecosystem services / plant interactions (pollination & seed dispersal). Introduced worldwide, including Mediterranean region and Iberia; spread facilitated by climatic warming but may be limited in Britain by winter temperature. | ([Roura-Pascual *et al.*, 2010](#_ENREF_46), [Roura-Pascual *et al.*, 2011](#_ENREF_47), [Roura-Pascual *et al.*, 2004](#_ENREF_48), [Wetterer *et al.*, 2009](#_ENREF_58)) |
| 11-20 | *Mnemiopsis leidyi* | American comb jelly | Ctenophora: Lobata | Predator | M | North America and South America | SA | 5 | 5 | 4 | 100 | Voracious predator of zooplankton including fish larvae and eggs. This species has been responsible for serious impacts in the Black Sea. Threat to more enclosed waters, especially of lower salinity. | ([Faasse & Bayha, 2006](#_ENREF_22), [Finenko *et al.*, 2006](#_ENREF_23)) |
| 11-20 | *Nassella neesiana (Stipa neesiana)* | Chilean needle grass | Angiosperm: Poaceae | Primary producer | T | South America | **Orn** | 5 | 5 | 3 | 75 | Increasingly cultivated because of current popularity of ornamental grasses; EPPO moved it from Alert to Observation list in 2012 with the similar *N. tenuissima* (prefers drier climates) and *N. trichotoma* (warmer summers). | ([Bourdôt *et al.*, 2012](#_ENREF_10), [EPPO, 2013](#_ENREF_20)) |
| 11-20 | *Proterorhinus marmoratus* | tubenose goby | Actinopterygii: Perciformes | Predator | F | Ponto-Caspian | **SA** | 4 | 5 | 5 | 100 | Evidence of diet overlap with native fishes, but unlike round goby does not feed on zebra mussels. Established widely in The Netherlands. | ([French & Jude, 2001](#_ENREF_26)) |
| 11-20 | *Rapana venosa* | veined rapa whelk | Mollusca: Gastropoda | Predator | M | Asia (Pacific) | SA, Aq | 5 | 4 | 5 | 100 | Large predatory gastropod, consumes a range of ecologically and commercially important invertebrates. Currently in North Sea, France and Netherlands. Very serious impacts in the Black Sea. Only limitation is current climate regime in GB waters, which may limit larval survival. | ([ICES, 2004](#_ENREF_29), [Mann & Harding, 2000](#_ENREF_36)) |
| 21-30 | *Agrilus plannipennis* | Emerald ash borer | Insecta: Coleoptera: Buprestidae | Herbivore | T | Asia | **For,** Nat | 3 | 5 | 4 | 60 | One of most destructive forest pests in USA & Canada, attacking *Fraxinus* spp.; not yet arrived in Europe;. Kills trees in 2-5 yrs; can affect entire stands with consequent impact on forest composition, succession and biogeochemical cycling. | ([Flower *et al.*, 2013a](#_ENREF_24), [Flower *et al.*, 2013b](#_ENREF_25), [Sobek-Swant *et al.*, 2012](#_ENREF_49)) |
| 21-30 | *Celtodoryx ciocalyptoides* | a sponge | Porifera: Poecilosclerida | Omnivore | M | Pacific | SA, Aq | 5 | 4 | 3 | 60 | Suspension feeder growing into very extensive patches. Variety of growth forms depending on habitat. Grows on *Eunicella verrucosa* (a slow growing, fragile, habitat forming species) but effects not studied. Extensive epifauna/flora. | ([Henkel & Janussen, 2011](#_ENREF_28), [Perez *et al.*, 2006](#_ENREF_43), [van Soest *et al.*, 2007](#_ENREF_53)) |
| 21-30 | *Dryocosmus kuriphilus* | oriental chestnut gall wasp | Insecta: Hymenoptera: Cynipidae | Herbivore | T | China | **For,** Nat | 3 | 5 | 3 | 45 | Causes up to 70% loss of fruit yield and may cause death of trees; possible positive interaction with chestnut blight. First recorded in Italy in 2002, France in 2005; spreading at approx 8km year; Britain is climatically suitable. | ([Bernardo *et al.*, 2013](#_ENREF_6), [Prospero & Forster, 2011](#_ENREF_45)) |
| 21-30 | *Echinogammarus ischnus* | bald urchin shrimp | Crustacea: Gammaridea | Omnivore | F | Ponto-Caspian | **SA** | 5 | 5 | 3 | 75 | In the Great Lakes in North America it forms relatively high densities in zebra mussel-dominated habitats, despite its somewhat lower fecundity and shorter life span in comparison to the indigenous gammarid species. Other established invasive gammarids in GB (e.g. *Gammarus tigrinus, Dikerogammarus villosus*) may suppress the impacts of this omnivore in some systems. Established and widespread in The Netherlands. | ([Bij de Vaate *et al.*, 2002](#_ENREF_7)) |
| 21-30 | *Gyrodactylus salaris* | salmon fluke | Platyhelminthes: Trematoda | Parasite | F | Baltic | **HF**, Aq, SA | 5 | 4 | 4 | 80 | A very small trematode parasite (<1mm) which attaches to the outer body and gills of salmon. It damages the skin and this can lead to infections. It has led to significant mortality of young salmon in river catchments in Norway, northern Finland and the White Sea area. Most vulnerable region in GB is Scotland, although strict species-specific biosecurity measures reduce the likelihood of introduction. | ([Johnsen & Jensen, 1991](#_ENREF_31)) |
| 21-30 | *Microstegium vimineum* | Japanese stiltgrass | Angiosperm: Poaceae | Primary producer | T | Central and eastern Asia | **SC**, RM | 3 | 4 | 5 | 60 | Potentially a highly invasive species in GB but currently with a very restricted distribution in Europe. | ([Adams & Engelhardt, 2009](#_ENREF_1), [EPPO, 2013](#_ENREF_20), [Gibson *et al.*, 2002](#_ENREF_27)) |
| 21-30 | *Nyctereutes procyonoides* | raccoon dog | Mammalia: Carnivora | Predator | T | Eastern Asia (Vietnam to Russia) | Orn | 4 | 3 | 5 | 60 | Rare escape/release in GB (invariably as single individuals) but abundant and increasing in central and eastern Europe. Fecund, adaptable, and potential threat to game birds, wildfowl and as vector of disease. | ([Kauhala & Kowalczyk, 2011](#_ENREF_33), [Parrott *et al.*, 2008](#_ENREF_42)) |
| 21-30 | *Ocenebra inornata* | Japanese sting winkle | Mollusca: Gastropoda | Predator | M | Asia (Pacific) | SA, Aq | 5 | 4 | 4 | 80 | A predator of bivalves. Possibly already here. Identification confusion possible with European stingwinkle. (Considered a serious threat to oysters in France.) | ([Lützen *et al.*, 2011](#_ENREF_35)) |
| 21-30 | *Tamias sibiricus* | Siberian chipmunk | Mammalia: Rodentia | Omnivore | T | Northern Asia (Kazahkstan to Japan) | Orn | 5 | 4 | 4 | 80 | Predator of ground-nesting birds and potential to compete with native rodents. Regular escape from captivity, often multiple individuals; proven establishment and spread in western Europe. | ([Long, 2003](#_ENREF_34), [Parrott *et al.*, 2008](#_ENREF_42)) |
| 21-30 | *Gracilaria vermiculophylla* | rough agar weed | Rhodophyta: Gracilariaceae | Producer | M | Pacific | SA, Aq | 5 | 5 | 4 | 100 | May impact *Fucus vesiculosus* (a key intertidal algal species on GB shores) populations and other native algae by means of several complex interactions. Able to grow rapidly and colonise large areas and is highly tolerant of low salinities. Possible interactions with sea grass beds and saltmarsh habitat. Thought to be present on South Coast of England but not established. | ([Nejrup *et al.*, 2012](#_ENREF_38), [Thomsen *et al.*, 2009](#_ENREF_51), [Weinberger *et al.*, 2008](#_ENREF_57)) |

Adams SN, Engelhardt KaM (2009) Diversity declines in Microstegium vimineum (Japanese stiltgrass) patches. *Biological Conservation,* **142**, 1003-1010.

Baker R, Anderson H, Matthews-Berry S, Korycinska A (2012) Rapid Pest Risk Analysis for *Thaumetopoea pityocampa* (the Pine Processionary Moth). York, FERA.

Baker SJ, Hills D (2008) Escapes and introductions. In: *The Mammals of the British Isles: Handbook, 4th Edition* (eds Harris S, Yalden D) pp 780-794. Southampton, The Mammal Society.

Baldwin BS, Mayer MS, Dayton J *et al.* (2002) Comparative growth and feeding in zebra and quagga mussels (Dreissena polymorpha and Dreissena bugensis): implications for North American lakes. *Canadian Journal of Fisheries and Aquatic Sciences,* **59**, 680-694.

Battisti A, Stastny M, Netherer S, Robinet C, Schopf A, Roques A, Larsson S (2005) Expansion of geographic range in the pine processionary moth caused by increased winter temperatures. *Ecological Applications,* **15**, 2084-2096.

Bernardo U, Gebiola M, Ixiao Z, Zhu C-D, Pujade-Villar J, Viggiani G (2013) Description of *Synergus castaneus* n. sp. (Hymenoptera: Cynipidae:Synergini) Associated With an Unknown Gall on *Castanea* spp. (Fagaceae) in China. *Annals of the Entomological Society of America,* **106**, 437-446.

Bij De Vaate A, Jazdzewski K, Ketelaars HaM, Gollasch S, Van Der Velde G (2002) Geographical patterns in range extension of Ponto-Caspian macroinvertebrate species in Europe. *Canadian Journal of Fisheries and Aquatic Sciences,* **59**, 1159-1174.

Blair MJ, Mckay H, Musgrove AJ, Rehfisch MM (2000) Review of the status of introduced non-native waterbird species in the Agreement area of the African-Eurasian Waterbird Agreement. (ed Ornithology BTF), Thetford.

Bodis E, Nosek J, Oertel N, Toth B, Feher Z (2011) A comparative study of two *Corbicula* morphs (Bivalvia, Corbiculidae) inhabiting River Danube. *International Review of Hydrobiology,* **96**, 257-273.

Bourdôt GW, Lamoureaux SL, Watt MS, Manning LK, Kriticos DJ (2012) The potential global distribution of the invasive weed *Nassella neesiana* under current and future climates. *Biological Invasions,* **14**, 1545-1556.

Caño L, Campos JA, Garcia-Magro D, Herrera M (2013) Replacement of estuarine communities by an exotic shrub: distribution and invasion history of *Baccharis halimifolia* in Europe. *Biological Invasions,* **15**, 1183-1188.

Csl (2004) Asian Longhorn Beetles.

Dauvin J-C (2009) Establishment of the invasive Asian shore crab *Hemigrapsus sanguineus* (De Haan, 1835) (Crustacea: Brachyura: Grapsoidea) from the Cotentin Peninsular, Normandy, France. *Aquatic Invasions,* **4**, 467-472.

Dauvin J-C, Delhay J-B (2010) First record of *Hemigrapsus takanoi* (Crustacea: Decapoda: Grapsidae) on the western coast of northern Cotentin, Normandy, western English Channel. *Marine Biodiversity Records,* **3**, 1-3.

Dauvin J-C, Tous Rius A, Ruellet T (2009) Recent expansion of two invasive crab species *Hemigrapsus sanguineus* (de Haan, 1835) and *H. takanoi* Asakura and Watanabe 2005 along the Opal Coast, France. *Aquatic Invasions,* **4**, 451-465.

Eppo (1999) EPPO Datasheets on Quarantine Pests *Anoplophora glabripennis*.

Eppo (2004) Diagnostic protocols for regulated pests. *Bulletin OEPP/EPPO 34,* **34**, 155 –157.

Eppo (2007) *Vespa velutina*: a new invasive alien species found in France.

Eppo (2009) Mini data sheet: *Baccharis halimifolia* (Asteraceae).

Eppo (2013) EPPO List of Invasive Plants.

Etienne B, Vanderhoeven S, Van Landuyt W, Van Rossum F, Verloove F (2010) *Baccharis halimifolia* - Eastern baccharis. (ed Species BFOI), Brussels.

Faasse MA, Bayha KM (2006) The ctenophore *Mnemiopsis leidyi* A.Agassiz 1865 in coastal waters of the Netherlands: an unrecognized invasion? . *Aquatic Invasions,* **1**, 270-277.

Finenko GA, Kideys AE, Anninsky BE *et al.* (2006) Invasive ctenophore *Mnemiopsis leidyi* in the Caspian Sea: feeding, respiration, reproduction and predatory impact on the zooplankton community. *Marine Ecology Progress Series,* **314**, 171-185.

Flower CE, Knight KS, Gonzalez-Meler MA (2013a) Impacts of the emerald ash borer (*Agrilus planipennis* Fairmaire) induced ash (*Fraxinus* spp.) mortality on forest carbon cycling and successional dynamics in the eastern United States. *Biological Invasions,* **15**, 931-944.

Flower CE, Knight KS, Rebbeck J, Gonzalez-Meler MA (2013b) The relationship between the emerald ash borer (*Agrilus planipennis*) and ash (*Fraxinus* spp.) tree decline: Using visual canopy condition assessments and leaf isotope measurements to assess pest damage. *Forest Ecology and Management,* **303**, 143-147.

French JRP, Jude DJ (2001) Diets and diet overlap of nonindigenous gobies and small benthic native fishes co-inhabitating the St. Clair River, Michigan. *Journal of Great Lakes Research,* **27**, 300-311.

Gibson DJ, Spyreas G, Benedict J (2002) Life history of *Microstegium vimineum* (Poaceae), an invasive grass in southern Illinois. *Journal of the Torrey Botanical Society,* **129**, 207-219.

Henkel D, Janussen D (2011) Redescription and new records of *Celtodoryx ciocalyptoides* (Demospongiae: Poecilosclerida)-a sponge invader in the north east Atlantic Ocean of Asian origin? *Journal of the Marine Biological Association of the United Kingdom,* **91**, 347-355.

Ices (2004) Alien Species Alert: *Rapana Venosa* (veined whelk). In: *ICES Cooperative Research Report.* (eds Mann R, Occhipinti A, Harding JM) pp 14.

Jensen GC, Mcdonald PS, Armstrong DA (2002) East meets west: competitive interactions between green crab *Carcinus maenus*, and native and introduced shore crab *Hemigrapsus* spp. *Marine Ecology Progress Series,* **225**, 251-262.

Johnsen BO, Jensen AJ (1991) The *Gyrodactylus* story in Norway. *Aquaculture,* **98**, 289-302.

Jørstad KE, A-L. A, Farestveit E (2011) The Introduced American Lobster, *Homarus americanus* in Scandinavian Waters. In: *In the Wrong Place - Alien Marine Crustaceans: Distribution, Biology and Impacts, Invading Nature.* (eds Galil BS, Clark PF, Carlton JD) pp 625-638. Springer.

Kauhala K, Kowalczyk R (2011) Invasion of the raccoon dog *Nyctereutes procyonoides* in Europe: history of colonisation, features behind its success and threats to native fauna. *Current Zoology,* **57**, 584-598.

Long JL (2003) *Introduced Mammals of the World: their history, distribution and influence,* Oxford, CABI Publishing.

Lützen J, Faasse M, Gittenberger A, Glennen H, Hoffmann E (2011) The Japanese oyster drill *Ocinebrellus inornatus* (Récluz, 1851) (Mollusca, Gastropoda, Muricidae), introduced to the Limfjord, Denmark. *Aquatic Invasions,* **7**, 181–191.

Mann R, Harding JM (2000) Invasion of the North American Atlantic coast by a large predatory Asian mollusc. *Biological Invasions,* **2**, 7-22.

Marris G, Roy HE (2013) Asian hornet: mistaken identity. *Bee Craft,* **95**, 30-32.

Nejrup LB, Pedersen MF, J. V (2012) Grazer avoidance may explain the invasiveness of the red alga *Gracilaria vermiculophylla* in Scandinavian waters. *Marine Bioloy,* **159**, 1703–1712.

Nyari A, Ryall C, Peterson AT (2006) Global invasive potential of the house crow *Corvus splendens* based on ecological niche modelling. *Journal of Avian Biology,* **37**, 306-311.

Ottens G (2003) Background and development of the Dutch population of House Crows *Corvus splendens*. *Limosa,* **76**, 69-74.

Ottens G, Ryall C (2003) House Crows in the Netherlands and Europe. *Dutch Birding,* **25**, 312-319.

Parrott D, Roy S, Fletcher M (2008) The status of scarce non-native birds and mammals in England. Central Science Laboratory.

Perez T, Perrin B, Carteron S, Vacelet J, Boury-Esnault N (2006) *Celtodoryx girardae* gen. nov sp nov., a new sponge species (Poecilosclerida : Demospongiae) invading the Gulf of Morbihan (North East Atlantic, France). *Cahiers De Biologie Marine,* **47**, 205-214.

Preston CD, Croft JM (1997) *Aquatic plants in Britain and Ireland,* Colchester, Harley Books.

Prospero S, Forster B (2011) Chestnut gall wasp (*Dryocosmus kuriphilus*) infestations: new opportunities for the chestnut blight fungus *Cryphonectria parasitica*? *New Disease Reports,* **23**, 35.

Roura-Pascual N, Bas JM, Hui C (2010) The spread of the Argentine ant: environmental determinants and impacts on native ant communities. *Biological Invasions,* **12**, 2399-2412.

Roura-Pascual N, Hui C, Ikeda T *et al.* (2011) Relative roles of climatic suitability and anthropogenic influence in determining the pattern of spread in a global invader. *Proceedings of the National Academy of Sciences of the United States of America,* **108**, 220-225.

Roura-Pascual N, Suarez AV, Gomez C, Pons P, Touyama Y, Wild AL, Peterson AT (2004) Geographical potential of Argentine ants (*Linepithema humile* Mayr) in the face of global climate change. *Proceedings of the Royal Society B-Biological Sciences,* **271**, 2527-2534.

Sobek-Swant S, Kluza DA, Cuddington K, Lyons DB (2012) Potential distribution of emerald ash borer: What can we learn from ecological niche models using Maxent and GARP? *Forest Ecology and Management,* **281**, 23-31.

Stebbing P, Johnson P, Delahunty A, Clark PF, Mccollin T, Hale C, Clark S (2012) Reports of American lobsters, *Homarus americanus* (H. Milne Edwards, 1837) (Crustacea: Decapoda: Astacidea: Nephropoidea) in Great British waters. *BioInvasions Records,* **1**, 17-23.

Thomsen MS, Mcglathery KJ, Schwarzschild A, Silliman BR (2009) Distribution and ecological role of the non-native macroalga *Gracilaria vermiculophylla* in Virginia salt marshes. *Biological Invasions,* **11**, 2303–2316.

Thum RA, Lennon JT (2010) Comparative ecological niche models predictive the invasive spread of variable-leaf milfoil (*Myriophyllum heterophllum*) and its potential impact on closely related native species. *Biological Invasions,* **12**, 133-143.

Van Soest RWM, De Kluijver MJ, Van Bragt PH *et al.* (2007) Sponge invaders in Dutch coastal waters. *Journal of the Marine Biological Association of the United Kingdom,* **87**, 1733-1748.

Vanderploeg HA, Nalepa TF, Jude DJ *et al.* (2002) Dispersal and emerging ecological impacts of Ponto-Caspian species in the Laurentian Great Lakes. *Canadian Journal of Fisheries and Aquatic Sciences,* **59**, 1209-1228.

Villemant C, Barbet-Massin M, Perrard A, Muller F, Gargominy O, Jiguet F, Rome Q (2011) Predicting the invasion risk by the alien bee-hawking Yellow-legged hornet *Vespa velutina nigrithorax* across Europe and other continents with niche models. *Biological Conservation,* **144**, 2142-2150.

Ward JM, Ricciardi A (2007) Impacts of *Dreissena* invasions on benthic macroinvertebrate communities: a meta-analysis. *Diversity and Distributions,* **13**, 155-165.

Weinberger F, Buchholz B, Karez R, Wahl M (2008) The invasive red alga *Gracilaria vermiculophylla* in the Baltic Sea: adaptation to brackish water may compensate for light limitation. *Aquatic Biology,* **3**, 251–264.

Wetterer JK, Wild AL, Suarez AV, Roura-Pascual N, Espadaler X (2009) Worldwide spread of the Argentine ant, *Linepithema humile* (Hymenoptera: Formicidae). *Myrmecological News,* **12**, 187-194.

Yesou P, Clergeau P (2006) Sacred Ibis: a new invasive species in Europe. *Birding World,* **18**, 517-526.
