# Supplementary material for: Horizon scanning for invasive alien species with the potential to threaten biodiversity in Great Britain
Source: Glob Chang Biol. 2014 May 19;20(12):3859–71. doi: 10.1111/gcb.12603 (PMC4283593; doi:10.1111/gcb.12603)
Supplement: Supplementary file 2 — Table S2. Species ranked as posing a medium risk (ranked equally within 31–93) with respect to likelihood of arriving, establishing and having an impact on native biodiversity in Britain over the next 10 years. [file gcb0020-3859-sd2.docx]

**Table S2:** Species ranked as posing a medium risk (ranked equally within 31 - 93) with respect to likelihood of arriving, establishing and having an impact on native biodiversity in Britain over the next ten years.

| **Species** | **Common name** | **Taxonomic group** |
| --- | --- | --- |
| *Acacia dealbata* | mimosa, silver wattle | Angiosperm |
| *Acacia melanoxylon* | Australian blackwood | Angiosperm |
| *Acer rufinerve* | grey snake-bark maple | Angiosperm |
| *Acridotheres tristis* | common myna | Aves |
| *Aedes albopictus* | Tiger mosquito | Insecta |
| *Aglaothamnion halliae* | A red alga | Rhodophyta |
| *Akebia quinata* | chocolate Vine | Angiosperm |
| *Alopex lagopus* | Arctic fox | Mammalia |
| *Amelanchier spicata* | dwarf serviceberry | Angiosperm |
| *Andropogon virginicus* | broomsedge bluestem | Angiosperm |
| *Anodonta woodiana* | Chinese giant mussel | Mollusca |
| *Anoplophora chinensis* | Citrus longhorn beetle | Insecta |
| *Antithamnion pectinatum* | A red alga | Rhodophyta |
| *Aonyx cinerea* | short clawed otter | Mammalia |
| *Asterias amurensis* | Northern Pacific seastar | Echinodermata |
| *Bacopa monnieri* | Brahmi | Angiosperm |
| *Caspihalacarus hyrcanus* | A mite | Acarina |
| *Castor canadensis* | American beaver | Mammalia |
| *Caulerpa taxifolia* | killer alga | Chlorophyta |
| *Chelicorophium robustum* | An amphipod | Amphipoda |
| *Chelicorophium sowinskyi* | An amphipod | Amphipoda |
| *Chelydra serpentina* | snapping turtle | Reptilia |
| *Chrysemys picta* | Easter painted turtle | Reptilia |
| *Cynomys ludocianus* | black-tailed prairie dog | Mammalia |
| *Cyperus esculentus* | yellow nut sedge, chuffa sedge | Angiosperm |
| *Dikerogammarus bispinosus* | An amphipod | Amphipoda |
| *Echinogammarus warpachowskyi* | An amphipod | Amphipoda |
| *Fraxinus pennsylvannica* | green ash | Angiosperm |
| *Gambusia holbrooki* | Eastern mosquito fish | Chordata |
| *Halyomorpha halys* | Brown marmorated stink bug | Insecta |
| *Jaera istri* | An isopod | Isopoda |
| *Limnobium spongia* | American sponge plant; American frog's-bit | Angiosperm |
| *Limnomysis benedeni* | A mysid | Mysidacea |
| *Lithoglyphus naticoides* | A gastropod | Gastropoda |
| *Marenzelleria wireni* | red gilled worm | Polychaeta |
| *Marsupenaeus japonicus* | Kuruma prawn | Dendrobranchiata |
| *Megabalanus coccopoma* | titan acorn barnacle/ large pink barnacle | Cirripedia |
| *Megabalanus tintinnabulum* | sea tulip | Cirripedia |
| *Mephitis mephitis* | striped skunk | Mammalia |
| *Myocaster coypus* | Coypu | Mammalia |
| *Mytilicola orientalis* | A parasitic copepod | Poecilostomatoida |
| *Nasella trichotoma* | serrated tussock | Angiosperm |
| *Nasua nasua* | coatimundi | Mammalia |
| *Neocaridina heteropoda* | A decapod | Decapoda |
| *Obesogammarus crassus* | An amphipod | Amphipoda |
| *Obesogammarus obesus* | An amphipod | Amphipoda |
| *Ondatra zibethicus* | muskrat | Mammalia |
| *Persicaria perfoliata* | Asiatic tearthumb, mile-a-minute weed | Angiosperm |
| *Pontogammarus robustoides* | An amphipod | Amphipoda |
| *Procambarus fallax* | marbled crayfish | Decapoda |
| *Pterois volitans* | red lionfish | Actinopterygii |
| *Pueraria montana var. lobata* | kudzu | Angiosperm |
| *Salamandra salamandra* | fire salamander | Amphibia |
| *Saururus cernuus* | swamp lily | Angiosperm |
| *Schizoporella errata* | A bryozoan | Bryozoa |
| *Solidago nemoralis* | grey goldenrod | Angiosperm |
| *Tadorna ferruginea* | ruddy shelduck | Aves |
| *Tamarix ramosissima* | salt cedar | Angiosperm |
| *Tamias striatus* | Eastern chipmunk | Mammalia |
| *Trapa natans* | water chestnut | Angiosperm |
| *Triadica sebifera* | candle-berry Tree | Angiosperm |
| *Zostera japonica* | Japanese seagrass | Angiosperm |
